# Summary: 2021 International Consultation on Incontinence Evidence-Based Surgical Pathway for Pelvic Organ Prolapse

**DOI:** 10.3390/jcm11206106

**Published:** 2022-10-17

**Authors:** Renaud de Tayrac, Danielle D. Antosh, Kaven Baessler, Cecilia Cheon, Xavier Deffieux, Robert Gutman, Joseph Lee, Charles Nager, Alexis Schizas, Vivian Sung, Christopher Maher

**Affiliations:** 1 Service de Gynécologie-Obstétrique, CHU de Nîmes, Université de Montpellier, 34000 Nîmes, France; 2 Department of Obstetrics and Gynecology, Houston Methodist Hospital, Houston, TX 77030, USA; 3 Pelvic Floor Centre, Franziskus and St. Joseph Hospitals, Budapester Str. 15-19, 10787 Berlin, Germany; 4 Urogynaecology Section of Queen Elizabeth Hospital, Hong Kong and Shenzhen Hong Kong University Hospital, Shenzhen 518009, China; 5 Service de Gynécologie-Obstétrique, Hôpital Antoine Béclère, 92140 Clamart, France; 6 Urogynecology & Pelvic Reconstructive Surgery, MedStar Washington Hospital Center, 106 Irving St. NW 405 S, Washington, DC 20010, USA; 7 St. Vincents Clinic, UNSW University of New South Wales, Sydney, NSW 2010, Australia; 8 Department of Obstetrics, Gynecology and Reproductive Sciences, University of California San Diego Health, San Diego, CA 92037, USA; 9 General Surgery Department, Guy’s and St. Thomas’ NHS Foundation Trust, Monkton Street, London SE11 4TX, UK; 10 Department of Obstetrics and Gynecology, The Warren Alpert Medical School of Brown University, Providence, RI 02903, USA; 11 Royal Brisbane and Womens Hospital, Urogynaecology University QLD, Herston, QLD 4029, Australia

**Keywords:** POP, pelvic organ prolapse, guideline, repair, mesh

## Abstract

(1) Background: There is wide variation in the reported prevalence rates for pelvic organ prolapse (POP). There is also wide variation in the rate at which surgical interventions for pelvic organ prolapse are performed, as well as the type of interventions undertaken. As part of the International Consultation on Incontinence (ICI), our committee was tasked to produce evidence-based pathways for the surgical management of POP, any associated stress urinary incontinence (SUI), and bowel dysfunction. (2) Methods: To enable us to generate such evidence, we undertook a thorough search for the POP surgery-related, English-language scientific literature published up to April 2021. (3) Results: The committee evaluated the literature and made recommendations based on the Oxford grading system. (4) Conclusions: This review serves to provide a summary of the 2021 ICI surgical management of an evidence-based prolapse pathway and outline the evidence used to inform this guidance.

## 1. Introduction

Pelvic organ prolapse (POP) is a common problem reported to affect up to 50% of parous women, and of these 62% would have undergone a surgical correction for this problem by the age of 80 [1,2,3]. Nonetheless, there is wide variation in the reported POP prevalence rates. Where POP is defined, and graded by symptoms, the prevalence is reported to be 3–6%, compared to 41–50% when based on examination [4,5,6]. On examination, anterior compartment prolapse is the most frequently reported site of prolapse and is detected twice as often as posterior compartmental defects and three times more commonly than apical prolapse [7,8]. Following hysterectomy, 6–12% of women will develop a vaginal vault prolapse and in two-thirds of these cases a multi-compartment prolapse is present [9,10,11].

There is wide variation in the rate at which surgical interventions for pelvic organ prolapse are performed in the USA (2.6/1000 women) compared to Switzerland (0.5/1000 women) [12]. There is also very significant variation in the type of interventions undertaken. Such large variations in the rate and types of surgery performed for pelvic organ prolapse may be explained by a variety of factors including women’s preferences, cultural and demographic variables, access to healthcare professionals, health professionals’ training, and a lack of a clear consensus regarding the guidelines for the surgical management of prolapses. Given the increasing time and resources that will be required for POP surgery in the future, it is paramount that we perform effective, durable, and cost-effective interventions with minimal morbidity. Furthermore, the variation in maternity practices, particularly with regard to operative vaginal delivery rates and types and the demographic changes such as an aging population, have significant implications for the future planning of women’s health services. However, this must be balanced against models that evaluate the impact of decreasing parity and increasing elective caesarean section rates are required to more accurately predict future rates of POP.

As part of the International Consultation on Incontinence (ICI), our committee was tasked to produce evidence-based pathways for the surgical management of POP, any associated stress urinary incontinence (SUI), and bowel dysfunction. To enable us to generate such evidence, we undertook a thorough search for the POP surgery-related English-language scientific literature on PubMed, Medline, the Cochrane library, and the Cochrane database of systematic reviews, published up to April 2021. The committee evaluated the literature and made recommendations based on the Oxford grading system, summarized as follows: a Grade A recommendation (GoR A), which usually depends on consistent randomized controlled trials (RCTs) or systematic reviews of RCTs; a Grade B recommendation (GoR B), which usually depends on consistent data from poor quality RCTs or prospective cohort studies or ‘majority evidence’ from RCTs; a Grade C recommendation (GoR C), which usually depends on retrospective or case series or Delphi-processed expert opinion; and a Grade D recommendation (GoR D)—‘No recommendation possible’ would be used where the evidence is inadequate or conflicting. This review serves to provide a summary of the 2021 ICI surgical management of the evidence-based prolapse pathway and outline the evidence used to inform this guidance.

## 2. POP Outcomes’ Assessment

POP is a multidimensional phenomenon, and the “success” of its treatment is often difficult to define. Historically, most studies focused exclusively on anatomical success without reporting the outcomes considered more important for women [13]. The Pelvic Organ Prolapse Quantification system (POPQ), despite its limitations, provides a reproducible and reliable description of the support of the anterior, posterior, and apical vaginal segments using precise measurements to a fixed reference point [14]. Reoperation for recurrence after POP surgery is an important measure of a procedure’s efficacy. In order to provide some clarity for future studies reporting reoperation rates after POP surgery, ICS/IUGA proposed a standardized definition for the different types of POP surgery [15]. Another important clinical outcome relates to the pre-operative complications of mesh and native tissue repairs and there are standardized systems for reporting these, which include the IUGA/ICS classification system for prosthesis/graft complication [16] or the Clavien–Dindo classification [17]. The reporting of these as individual rather than composite outcome measures allows for more ready and reliable comparisons in meta-analyses.

In contrast, patient-reported outcomes (PRO) or outcome measures (PROM) are tools used to measure any aspect of a patient’s health status that comes directly from the patient. These can be generic and condition-specific. Generic PRO instruments are used to assess the health-related quality of life (HRQOL) status in a broad range of illnesses or populations while condition-specific measures are designed to measure the impact of a specific disease. The evaluation of a patient with a vaginal prolapse requires a comprehensive review of the full spectrum of pelvic floor symptoms and their impacts on the woman’s quality of life. A symptom that is almost consistently acknowledged by patients with advanced POP is the presence of a vaginal bulge that can be seen or felt [6,18,19]. ICS/IUGA also recommend reporting the sexually functional status of all individual participants pre- and post-intervention [15]. Moreover, assessing the patient’s satisfaction is important yet complex because the determinants of satisfaction are multifactorial. The patient global impression of severity (PGI-S) or improvement (PGI-I) is often used as the validated PRO to measure improvement and satisfaction following prolapse treatment [20].

Based on reviewing the literature relating to POP outcome measures, our committee’s recommendations are as follows:

- The anatomical outcomes reported should include all POP-Q points and staging utilizing a traditional definition of success. Assessments should be prospective, and assessors should be blinded to the surgical intervention performed wherever possible and should not possess any conflict of interest related to the assessment undertaken (GoR C).
- Prolapse surgery should be defined as primary surgery and repeat surgery sub-classified as primary surgery of a different site for repeat surgeries, complications related to surgery, and surgery for non-prolapse-related conditions (GoR C).
- Functional outcomes are best reported using valid, reliable, and responsive symptom questionnaires and condition-specific HRQOL instruments (GoR C).
- Sexual function is best reported utilizing validated condition-specific HRQOL instruments that assess sexual function or validated sexual function questionnaires such as the Pelvic Organ Prolapse/Incontinence Sexual Questionnaire (PISQ) or the Female Sexual Function Index (FSFI). The sexual activity status of all the study participants should be reported pre-and post-operatively under the following categories: sexually active without pain, sexually active with pain, or not sexually active (GoR C).

## 3. Anterior Compartment Surgery

Historically, anterior colporrhaphy (AC) was the standard procedure in the management of an anterior compartment prolapse. The importance of paravaginal defects in anterior compartment prolapses has been highlighted since 1912 [21]. Since then, several vaginal, abdominal, and laparoscopic procedures have been described. However, no randomized control studies have evaluated these in isolation. Raz et al. (96) popularized the needle suspension type procedure for cystocele repair and reported success rates in case series ranging from 90 to 98% [22,23]. Further studies demonstrated a reduction in recurrence and reoperation rates when an apical suspension procedure is performed [24,25,26]. Nevertheless, this message does not seem to be reflected in clinical practice. 

Following high reported objective failure and reoperation rates after native tissue repairs, the last decade has seen both a rapid uptake and subsequent decline in the use of transvaginal permanent meshes for the management of anterior compartment prolapse. A Cochrane review, evaluating nearly 2000 women, demonstrated some advantages and disadvantages to the use of polypropylene mesh in anterior compartment surgery [27]. More recently, the majority of the polypropylene mesh products evaluated in this meta-analysis have been voluntarily withdrawn by the manufacturers in the face of ongoing litigation. Pain and dyspareunia were the leading causes of adverse events that triggered the 2011 FDA warnings on the safety of transvaginal meshes [28,29]. Absorbable meshes were proposed as an attractive option because of the proposed increased strength during the early healing phase without the long-term complications of a permanent mesh. Nevertheless, they seem to offer little advantage compared to AC for anterior compartment prolapse [27]. Furthermore, autologous grafts and allografts have been tested to improve the outcomes of native tissue repair while mitigating the risks associated with synthetic meshes. However, a meta-analysis of eight trials using various biological grafts demonstrated that they had similar outcomes to AC regarding prolapse awareness and reoperation [27].

Transvaginal meshes have also been shown to have some advantages in women with a recurrent anterior prolapse, yet with a similar risk profile to that described for primary repairs and even higher mesh exposure rates. Although some clinicians believe that polypropylene mesh still has a role in recurrent prolapse, the number of proponents of such a policy is declining. 

Further, with respect to the review of the current evidence, our current committee’s recommendations for the management of anterior compartment POP are as follows:

- Polypropylene mesh has a superior anatomical outcome compared to a biological graft (Pelvicol) in the anterior compartment (Feldner, Menefee). However, the mesh exposure rate was significantly higher in the polypropylene mesh group (GoR A).
- Polypropylene mesh demonstrates improved anatomical and subjective outcomes compared to AC with no difference in the functional outcomes using validated questionnaires or a lower reoperation rate for prolapse. The mesh group was also associated with longer operating times, greater blood loss, and a non-significant tendency towards higher cystotomy, de novo dyspareunia, and de novo stress urinary incontinence rates compared to AC. Apical or posterior compartment prolapse was significantly more common following polypropylene mesh and the mesh exposure rate was 10.4% with 6.3% undergoing surgical correction (GoR B).
- The data for recurrent anterior vaginal wall prolapses show conflicting information regarding the advantages for polypropylene mesh compared to AC, with relatively high rates of mesh complication reported in the long-term (GoR C).

## 4. Surgical Treatment of Uterovaginal Prolapse

Despite the fact that most gynecologists and pelvic-reconstructive surgeons consider the uterus to be a passive structure in prolapse development, it is frequently removed during uterovaginal prolapse surgery. Surveys of women suggest that 40–60% of respondents in the USA, the Netherlands, Austria/Germany, and Russia would choose uterine preservation assuming equal surgical efficacy [30,31,32,33]. In contrast, 66% of female gynecologists in the Czech Republic, Slovenia, and Slovakia preferred concomitant hysterectomy if they were to have a POP that required a repair assuming equal outcomes [34].

Due to the limited data regarding the risks associated with subsequent pregnancy and delivery, surgery should be reserved for those that cannot be managed with a pessary. This review focuses on women who have completed childbearing and are postmenopausal or practicing reliable contraception with a particular focus on comparing hysteropexy and hysterectomy surgical outcomes for the treatment of uterovaginal prolapses. Moreover, the candidates for uterine conservation should be made aware that if a subsequent hysterectomy is required, it could be more technically challenging. Women at increased risk for endometrial, cervical, or ovarian cancer and those with a personal history of estrogen receptor-positive breast cancer, especially those taking Tamoxifen, should have their uterus, cervix, and possibly ovaries removed at the time of the prolapse repair. 

Broadly, there are different types of native tissue repairs involving uterine conservation: LeFort colpocleisis, the Manchester procedure, Sacrospinous hysteropexy (SSHP), and uterosacral hysteropexy (USHP). Whereas there are two main types of mesh hysteropexy procedures: SSHP with a graft, previously referred to as vaginal mesh hysteropexy, and sacrohysteropexy (SHP). Regarding SSHP with a graft, all of the first and second generation anterior and posterior mesh kits are no longer commercially available. SHP typically involves the attachment of at least one graft from the cervix and uterus to the anterior longitudinal ligament near the sacral promontory. This is an abdominal procedure that can be performed via an open, laparoscopic, or robotic approach. A variety of graft materials, configurations, and operative techniques have been described, which may explain the differences in anterior wall recurrences and the development of cervical elongation. Supracervical hysterectomy and sacrocervicopexy (SCerP) were introduced to obtain the benefits of SCP and hysterectomy without the risk of mesh exposure. However, the data to support or refute this claim are still awaited.

In summary, there are numerous options for the primary treatment of uterovaginal prolapse. The committee’s evidence-based recommendations regarding the management of uterovaginal prolapse and uterine preservation are as follows:

- Hysteropexy is reasonable in women undergoing surgery for uterovaginal prolapse without contraindications to uterine preservation. However, long-term data are limited and the need for subsequent hysterectomy is unknown (GoR C).
- When considering relative contra-indications to uterine preservation, an opportunistic salpingectomy, which cannot be performed during vaginal hysteropexy, should be included in the shared decision-making process (GoR C).
- Large database studies demonstrated lower reoperation rates for recurrent prolapses and slightly higher complication rates in the hysterectomy group compared to hysteropexy (GoR C).
- Consistent level one and two evidence reveal no differences in outcomes comparing sacrospinous hysteropexy to vaginal hysterectomy with native tissue prolapse repair, with the exception of a single smaller RCT showing a higher risk of apical recurrence for patients with advanced prolapse undergoing hysteropexy (GoR B).
- A partial Colpocleisis is preferred over a vaginal hysterectomy and total colpocleisis when there is no specific indication for hysterectomy and no interest in preserving coital function (GoR C).
- The role of the Manchester procedure for the treatment of a mild uterovaginal prolapse with or without cervical elongation has yet to be determined based on limited, poor level two and three evidence (GoR D).
- The data are not supportive of transvaginal meshes and hysterectomy for uterine prolapse. Consistent Level two evidence shows no difference in anatomic success when comparing a sacrospinous hysteropexy with a mesh graft to a hysterectomy with a graft; additionally, the mesh exposure rate was significantly higher after a hysterectomy than hysteropexy (11% vs. 5%) (GoR C).
- Sacrohysteropexy (SHP) has a similar success rate and reoperation for prolapse when compared to a vaginal hysterectomy and USLS; however, it has lower success rates than sacrocolpopexy with total or supracervical hysterectomy (GoR C).
- A sacrocolpopexy with a total hysterectomy is not recommended due to a three- to five-fold higher mesh exposure rate (GoR C).
- A single small study sacrocolpopexy with a supracervical hysterectomy had a lower anatomic success rate than a sacrocolpopexy with a total hysterectomy (GoR D)
- Level three evidence reveals low rates of unanticipated pathology (1.5%) and endometrial cancer (0.3%) with no cases of sarcoma identified during hysterectomies in women with a low risk of malignancy and dysplasia undergoing prolapse surgery (GoR C).

## 5. Apical Prolapse Surgery

A loss of apical support is usually present in women with a prolapse that extends beyond the hymen [35,36]. There is growing recognition that adequate support for the vaginal apex is an essential component of a durable surgical repair for women with an advanced prolapse [37,38]. Apical suspension procedures can be classified into those performed transvaginally and those performed abdominally. Abdominal procedures, predominantly sacrocolpopexy, can be performed via laparotomy or using conventional laparoscopic or robotically assisted-laparoscopic techniques. Transvaginal apical suspension procedures include both non-mesh (native tissue) procedures and mesh repairs. 

Sacrospinous ligament fixation (SSLF) is one of the most popular and widely reported native tissue transvaginal procedures for correcting apical prolapses. Ipsilateral uterosacral ligament fixation (USLS) suspends the vaginal apex to the proximal remnants of the uterosacral ligaments using an intraperitoneal surgical approach. This procedure restores the vagina to a near normal axis, avoiding the greater occurrence of retroflexion associated with SSLF. Traditionally, sacrocolpopexies have been performed via a laparotomy (i.e., an abdominal sacrocolpopexy, ASCP) but the use of minimally invasive approaches, both laparoscopic (LSCP) and robotic (RSCP), has become the norm over the last decade. Obliterative surgery, such as total or the LeFort partial colpocleisis, can also be used in the management of apical POP, but are usually reserved for women who are elderly, medically compromised, and no longer sexually active.

In addition to the above synthesis of the evidence, the committee’s conclusions regarding apical prolapse surgery are as follows:

- A single large RCT suggests that USLS and SSLF have similar anatomical, functional, and adverse event outcomes (GoR B).
- Level one evidence demonstrates transvaginal mesh procedures offer no significant advantage over vaginal native tissue apical repairs and are associated with mesh exposures (GoR A).
- Level one evidence suggests that overall sacrocolpopexy is associated with a lower risk of awareness of prolapse, a recurrent prolapse on examination, repeat surgery for prolapse, and post-operative SUI and dyspareunia when compared broadly with vaginal prolapse repairs with and without mesh augmentation (GoR A).
- Level one evidence suggests that ASCP has a higher success rate as compared to SSLS with fewer occurrences of SUI and post-operative dyspareunia. ASC had a greater morbidity including regarding operating time, inpatient stay, a slower return to activities of daily living, and a higher cost (GoR A).
- In a single RCT, ASCP was associated with greater anatomical success, fewer reoperations, and greater post-operative complications than USLS but no difference in the improvement in symptoms or quality of life was reported (GoR B).
- LSCP is associated with lower levels of blood loss, longer operating times, and shorter hospital stays than ASCP, with no difference in the objective or subjective cure rates (GoR B).
- Compared to LSCP, RSCP is associated with longer operating times, greater post-operative pain, and a higher cost with similar rates of anatomic success and complications (GoR B).
- ASCP performed with polypropylene mesh has superior outcomes to fascia lata (GoR B).
- In a single RCT, LSCP had superior objective and subjective success rates and lower reoperation rates compared to polypropylene transvaginal mesh for vault prolapses (GoR B).
- Level three evidence suggests McCall culdoplasty, Iliococcygeus fixation, and colpocleisis are relatively safe and effective interventions (GoR C).

## 6. Surgery for Posterior Vaginal Wall Prolapse

Based on the work of DeLancey [39], the connective tissue support of the vagina can be divided into three levels: the apical portion of the posterior vagina (level I), supported primarily by the cardinal-uterosacral ligaments; the mid-section of the vagina (level II), supported by the endopelvic fascia; and the distal support of the posterior vaginal wall (level III), which is primarily provided by the perineal body.

The prolapse of the posterior vaginal wall may be secondary to the presence of rectocele, sigmoidocele, enterocoele, or a combination of these. A posterior vaginal prolapse can be associated with a bothersome vaginal bulge as well as emotional, sexual, and defecatory dysfunctions. Its surgical treatment should be primarily driven by the patient’s symptoms and discomfort. Of note, many patients may present with both defecatory symptoms as well as a posterior vaginal prolapse leading to the assumption that the prolapse is causing the problems. However, the data are conflicting regarding the efficacy of posterior vaginal repair for improving defecatory symptoms and the association is incompletely understood [40,41]. The types of surgical repair for a posterior vaginal prolapse include midline plication (native tissue), site-specific techniques (native tissue), graft/mesh augmentation of the midline or site-specific repairs, transanal repair, ventral rectopexy, and sacral colpopexy, in which the mesh is extended to the distal portion of the posterior vaginal wall and/or perineum. 

**Figure 1:**
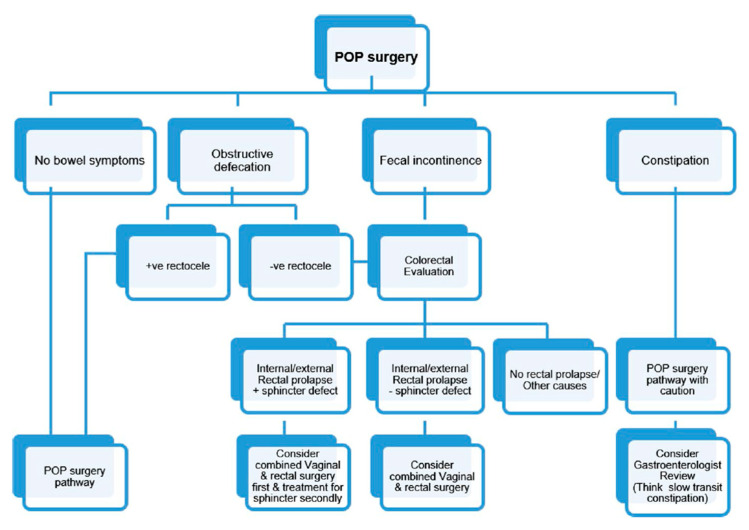
Suggested surgical pathway for prolapse repair with coexisting bowel symptoms.

Women with a vaginal prolapse frequently have a coexistent bowel dysfunction including obstructed defection, constipation, and fecal incontinence. We were unable to identify an evidence-based pathway for the management of pelvic organ prolapse and associated bowel symptoms. So, utilizing the wide clinical and academic experience of the committee members and the Delphi process, we established clinical guidance for the management of women with a vaginal prolapse and associated bowel symptoms as seen in Figure 1.

The current committee’s recommendations for the surgery of the posterior vaginal wall prolapse are as follows:

- Level one and two evidence suggest that a midline plication posterior repair without a levatorplasty has superior objective outcomes as compared to a site-specific posterior repair (GoR B).
- A higher dyspareunia rate is reported when a levatorplasty is performed (GoR C).
- The transvaginal approach is superior to the transanal approach for the repair of a posterior wall prolapse (GoR A).
- To date, no study has shown any benefit to a graft or mesh overlay or to the augmentation of a suture repair for a posterior vaginal wall prolapse (GoR B).
- While modified abdominal sacrocolpopexy results have been reported, the data on how these results would compare to the traditional transvaginal repair of a posterior vaginal wall prolapse are lacking.
- The data comparing Delorme’s procedure and ventral mesh rectopexy (VMR) for an external rectal prolapse are conflicting, with a single RCT demonstrating no statistical difference, while the level 3 data are supportive of VMR performed laparoscopically or robotically, with low rates of recurrent rectal prolapses and improved rates of fecal incontinence and constipation (GoR D).
- VMR appears superior to other abdominal rectopexies (posterior mesh rectopexy, Ripstein, and Orr–Loygue) with different rectal mobilizations to treat ERP in terms of functional outcome (GoE C).
- LoE 3 supports a ventral rectopexy for an Oxford grade 3–4 internal rectal prolapse. The data are not conclusive regarding the graft material or route of surgery (GoR C).
- No data demonstrate that a ventral rectopexy with or without a graft attachment to the posterior vaginal wall is effective for the management of rectocele (GoR D).
- Limited level three evidence suggest that patients with combined rectal and vaginal prolapses benefit from colorectal surgeons and urogynecologist collaborating closely (GoR C).

## 7. Surgery for Pelvic Organ Prolapse and Bladder Function

Patients with POP often present with bladder symptoms such as urinary incontinence or voiding difficulties. Many women with advanced POP do not experience SUI. However, if the prolapse is reduced digitally or with the help of a pessary, sponge holder, or speculum, SUI may be demonstrated in 10 to 80% [42,43,44,45]. This type of SUI, which becomes only obvious with the prolapse reduced in otherwise continent women, is termed an occult, masked, or latent SUI. Although different techniques to reduce the prolapse have been described, a gold-standard has not been established [46,47]. Neither the speculum nor the pessary test to reduce the prolapse had acceptable positive predictive values to identify women in need of a concomitant continence procedure. However, women with preoperatively negative tests for occult SUI are at low risk of developing SUI postoperatively [46,48]. 

**Figure 2:**
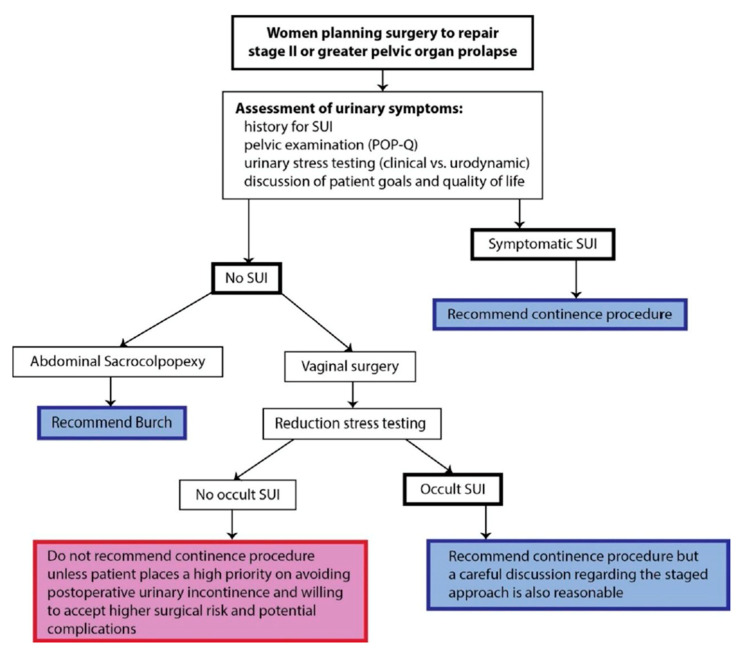
Decision-making flowchart for women undergoing prolapse surgery with or without SUI.

Our review group have evaluated the current evidence relating to women undergoing prolapse surgery with or without SUI and devised a decision-making flowchart (Figure 2) together with the following recommendations:

- Continent women who test negative for occult SUI do not require a concurrent prophylactic continence procedure (GoR B).
- In continent women who test negative for occult SUI undergoing sacrocolpopexy, an additional Burch colposuspension may reduce the occurrence of postoperative SUI (GoR C).
- Anterior mesh repair increases the risk for SUI as compared to anterior repair without mesh in continent women (GoR B).
- Continent women with occult SUI benefit from POP surgery with concomitant continence procedures as compared to POP surgery without continence intervention (GoR B).
- In women with POP and SUI, a concomitant continence procedure increases postoperative SUI cure rates (GoR A).
- A preoperative OAB (40%) resolves in approximately 50% of post-prolapse surgeries, although the impact of a concomitant non- surgical treatment remains unclear (GoR C).
- The rate of de novo OAB varies widely (2–32%) (GoR C).
- The rates of urinary retention following POP surgery varies from 0–34% and are nearly always temporary (GoR C).
- Pre-operative urinary retention resolves in as many as 90% of post-prolapse surgeries (GoR C).

## 8. Pelvic Organ Prolapse Surgery and Sexual Function

The prevalence of sexual dysfunction in patients with pelvic organ prolapse (POP) and other pelvic floor disorders is approximately 64% [48]. Therefore, it is important to assess the sexual function in patients undergoing reconstructive surgery for a prolapse in general practice and research trials. According to the IUGA-ICS Joint Report on the Terminology and Assessment of the Sexual Health of Women with Pelvic Floor Disorders, trials should report both the pre-operative AND post-operative outcomes for sexual activity, dyspareunia, and validated sexual function questionnaire scores [49]. The validated measures for sexual function in relation to POP surgery have been discussed above. The summary of our comprehensive review of different types of POP surgery and perioperative sexual function is as follows:

- While synthetic transvaginal mesh and non-mesh vaginal repairs have similar rates of de novo and total dyspareunia, the transvaginal mesh repair has a poorer sexual function as measured by the PISQ when compared to non-mesh repairs (GoR B).
- Synthetic transvaginal meshes have a higher rate of total dyspareunia when compared to sacrocolpopexy (GoR B).
- When comparing vaginal biologic grafts to vaginal native tissue repairs, there are similar decreases in postoperative dyspareunia and similar changes in sexual function (GoR B).
- Postoperative sexual activity in patients undergoing POP reconstructive surgery ranges from 42–65%, while postoperative sexual activity ranges from 32–62% (GoR C).
- The de novo dyspareunia rates for native tissue vaginal repair and sacrocolpopexy range from 2–8% (GoR C).
- Total dyspareunia rates generally decrease following native tissue repairs and sacrocolpopexy from 15–30% preoperatively to 8–20% postoperatively (GoR C).
- It is preferable to use validated questionnaires measuring sexual function in women before and after prolapse surgery. We also recommend reporting sexual activity and dyspareunia rates de novo, pre-, and post-intervention in all patients (GoR C).

## 9. Discussion

**Figure 3:**
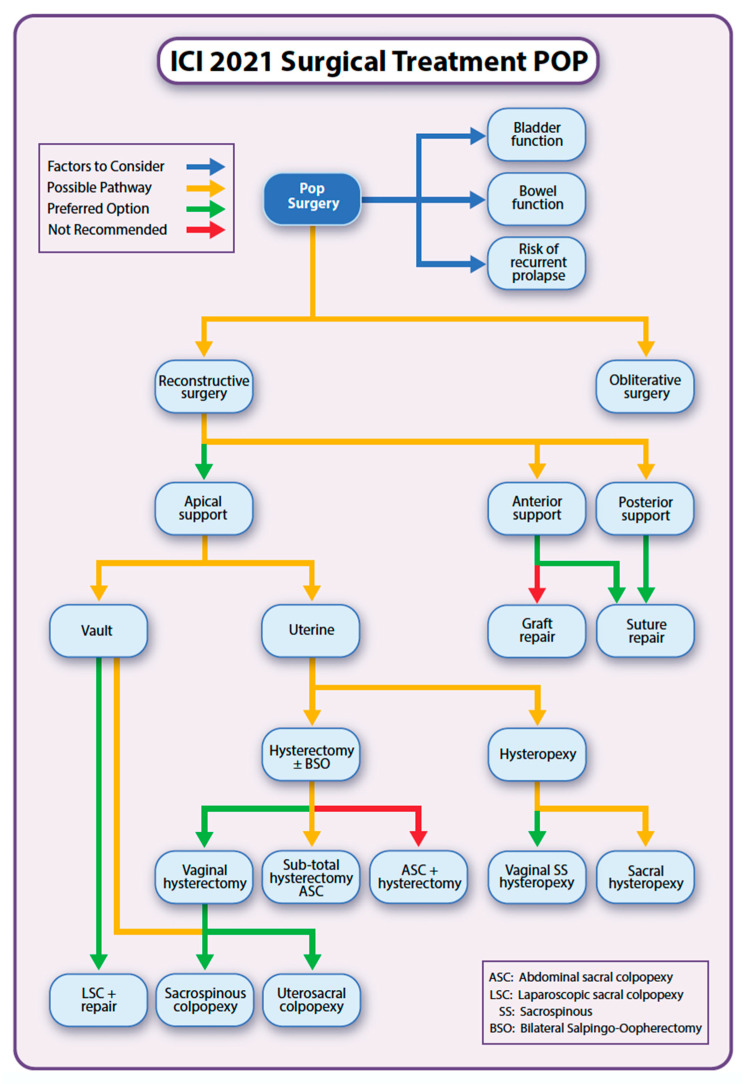
ICI 2021 pathways for surgical treatment of POP.

Despite our robust methodology and thorough review of the literature relating to the surgical management of POP, we have not identified consistent evidence for the superiority of a single intervention that has resulted in better outcomes compared to others as assessed by validated quality of life pelvic floor questionnaires. This suggests that either the interventions are not significantly different between women or that the questionnaires are not sensitive enough to detect a change. Notwithstanding this problem, our committee has developed a treatment algorithm for the surgical management of prolapses (Figure 3). 

Similar to the 2017 pathway [50], the 2021 ICI pathway for the surgical management of prolapse utilizes traffic light-colored arrows to guide treatment options, with a green line indicating the preferred option, yellow a possible alternative, and red indicating that further data are required to make a comprehensive assessment and, hence, that this treatment option is currently not recommended. The decisions that informed the algorithm are based on the findings in the report and the recommendations summarized above. Nonetheless, our committee recognizes that the algorithm is a guideline for both patients and clinicians and that patients’ treatment is best individualized and underpinned by an open and transparent consultation process and consent.

Our review has also demonstrated that as a group we continue to perform surgeries with limited evidence to support their safety and efficacy. One example relates to several of the newer lightweight mesh devices that are currently available. Indeed, these products require a significant further evaluation prior to being introduced into treatment pathways for the surgical management of prolapses. A second example relates to the anecdotal perception that the performance of a sacral colpopexy for uterine prolapse is increasingly common. The confidence in surgical outcomes relating to sacral colpopexy is largely derived from post-hysterectomy prolapse data. Interestingly, while there are increasing data to support the performance of a sacral colpopexy for a post-hysterectomy prolapse, the limited early data available in our review suggest that neither sacrohysteropexy nor supracervical or total hysterectomy and sacral colpopexy are superior to a variety of vaginally based interventions. Hence, the current evidence-based algorithm points towards vaginally based native tissue interventions for primary uterine prolapses and reserving sacral colpopexy for post-hysterectomy and recurrent prolapses.

Many countries have prohibited access to vaginal meshes. However, in countries where transvaginal meshes are still available, patients should be made aware that a mesh is considered a permanent implant and that it might not be possible to completely remove it or reverse its complications despite multiple operations. Patients should be informed that transvaginal meshes have a higher reoperation rate than native tissue vaginal repairs (CoR A). Therefore, patients must be informed of conservative and alternative surgical techniques. If a synthetic mesh is placed by the vaginal route, it is recommended that a macroporous polypropylene monofilament mesh is used while the use of a polyester mesh is not recommended (GoR B). Moreover, it is our expert opinion that a non-absorbable synthetic mesh should not be inserted into the rectovaginal septum when a rectal injury occurs.

Regarding sacrocolpopexy, the use of silicone-coated polyester, porcine dermis, fascia lata, and polytetrafluoroethylene meshes are not currently recommended. Our review demonstrated that a laparoscopic approach, polyester (without silicone coating) or monofilament polypropylene meshes, and the use of delayed absorbable sutures for securing the mesh to the vagina are recommended (GoR C). Furthermore, a concomitant total hysterectomy seems to be associated with an increased risk of mesh exposure (GoR C). Finally, our group also recommends that the type and commercial name of mesh used is clearly recorded and that peritoneal closure is recommended to cover the meshes. 

Finally, there is wide variation and lack of consistency regarding non-procedure-related causes for recurrence; these include patients’ characteristics, a high versus low volume surgeons, and the role of a physiotherapist. Based on our review, there is current evidence to suggest that the rate of recurrence is associated with the patient’s age < 60 years, POP stages of 3–4, and low volume surgeons. In contrast, a poor levator strength and perioperative physiotherapist do not seem to increase or protect against recurrence, respectively. Whereas the evidence for the role of levator defects and enlarged levator hiatus in POP recurrence is conflicting [51,52,53,54,55,56].

## Data Availability

The literature searches and review data is available in the full guideline and upon request from the corresponding author.

## References

[1] Olsen A.L., Smith V.J., Bergstrom J.O., Colling J.C., Clark A.L. (1997). Epidemiology of surgically managed pelvic organ prolapse and urinary incontinence. Obstet. Gynecol..

[2] Smith F.J., Holman C.D.J., Moorin R., Tsokos N. (2010). Lifetime Risk of Undergoing Surgery for Pelvic Organ Prolapse. Obstet. Gynecol..

[3] Wu J.M., Matthews C.A., Conover M., Pate V., Funk M.J. (2014). Lifetime Risk of Stress Urinary Incontinence or Pelvic Organ Prolapse Surgery. Obstet. Gynecol..

[4] Phillips C., Anthony F., Benyon C., Monga A.K. (2005). Urogynaecology: Collagen metabolism in the uterosacral ligaments and vaginal skin of women with uterine prolapse. Int. J. Obstet. Gynaecol..

[5] Nygaard I. (2008). Prevalence of Symptomatic Pelvic Floor Disorders in US Women. J. Am. Med. Assoc..

[6] Swift S.E., Tate S.B., Nicholas J. (2003). Correlation of symptoms with degree of pelvic organ support in a general population of women: What is pelvic organ prolapse?. Am. J. Obstet. Gynecol..

[7] Handa V.L., Garrett E., Hendrix S., Gold E., Robbins J. (2004). Progression and remission of pelvic organ prolapse: A longitudinal study of menopausal women. Am. J. Obstet. Gynecol..

[8] Hendrix S.L., Clark A., Nygaard I., Aragaki A., Barnabei V., McTiernan A. (2002). Pelvic organ prolapse in the women’s health initiative: Gravity and gravidity. Am. J. Obstet. Gynecol..

[9] Aigmueller T., Dungl A., Hinterholzer S., Geiss I., Riss P. (2009). An estimation of the frequency of surgery for posthysterectomy vault prolapse. Int. Urogynecol. J..

[10] Morley G.W., DeLancey J.O. (1988). Sacrospinous ligament fixation for eversion of the vagina. Am. J. Obstet. Gynecol..

[11] Marchionni M., Bracco G.L., Checcucci V., Carabaneanu A., Coccia E.M., Mecacci F., Scarselli G. (1999). True incidence of vaginal vault prolapse. Thirteen years of experience. J. Reprod. Med..

[12] Haya N., Baessler K., Christmann-Schmid C., de Tayrac R., Dietz V., Guldberg R., Mascarenhas T., Nussler E., Ballard E., Ankardal M. (2015). Prolapse and continence surgery in countries of the Organization for Economic Cooperation and Development in 2012. Am. J. Obstet. Gynecol..

[13] Barber M.D., Brubaker L., Nygaard I., Wheeler T.L., Schaffer J., Chen Z., Spino C. (2009). Defining Success after Surgery for Pelvic Organ Prolapse. Obstet. Gynecol..

[14] Haylen B.T., Maher C.F., Barber M.D., Camargo S., Dandolu V., Digesu A., Goldman H.B., Huser M., Milani A.L., Moran P.A. (2016). An International Urogynecological Association (IUGA)/International Continence Society (ICS) joint report on the terminology for female pelvic organ prolapse (POP). Int. Urogynecol. J..

[15] Toozs-Hobson P., Freeman R., Barber M., Maher C., Haylen B., Athanasiou S., Swift S., Whitmore K., Ghoniem G., De Ridder D. (2012). An International Urogynecological Association (IUGA)/International Continence Society (ICS) joint report on the terminology for reporting outcomes of surgical procedures for pelvic organ prolapse. Int. Urogynecol. J..

[16] Haylen B.T., Freeman R.M., Swift S.E., Cosson M., Davila G.W., Deprest J., Dwyer P.L., Fatton B., Kocjancic E., Lee J. (2010). An international urogynecological association (IUGA)/international continence society (ICS) joint terminology and classification of the complications related directly to the insertion of prostheses (meshes, implants, tapes) and grafts in female pelvic flo. Neurourol. Urodyn..

[17] Dindo D., Demartines N., Clavien P.-A. (2004). Classification of Surgical Complications: A new proposal with evaluation in a cohort of 6336 patients and results of a survey. Ann. Surg..

[18] Ellerkmann R.M., Cundiff G.W., Melick C.F., Nihira M.A., Leffler K., Bent A.E. (2001). Correlation of symptoms with location and severity of pelvic organ prolapse. Am. J. Obstet. Gynecol..

[19] Barber M.D. (2005). Symptoms and Outcome Measures of Pelvic Organ Prolapse. Clin. Obstet. Gynecol..

[20] Srikrishna S., Robinson D., Cardozo L. (2009). Validation of the Patient Global Impression of Improvement (PGI-I) for urogenital prolapse. Int. Urogynecol. J..

[21] White G. (1912). An anatomic operation for the cure of cystocele. Am. J. Obstet. Dis. Women Child..

[22] Raz S., Klutke C.G., Golomb J. (1989). Four-Corner Bladder and Urethral Suspension for Moderate Cystocele. J. Urol..

[23] Raz S., Little N.A., Juma S., Sussman E.M. (1991). Repair of Severe Anterior Vaginal Wall Prolapse (Grade IV Cystourethrocele). J. Urol..

[24] Eilber K.S., Alperin M., Khan A., Wu N., Pashos C.L., Clemens J.Q., Anger J.T. (2013). Outcomes of Vaginal Prolapse Surgery among Female Medicare Beneficiaries. Obstet. Gynecol..

[25] Goldberg R.P., Koduri S., Lobel R.W., Culligan P.J., Tomezsko J.E., Winkler H.A., Sand P.K. (2001). Protective effect of suburethral slings on postoperative cystocele recurrence after reconstructive pelvic operation. Am. J. Obstet. Gynecol..

[26] Gardy M., Kozminski M., DeLancey J., Elkins T., McGuire E.J. (1991). Stress Incontinence and Cystoceles. J. Urol..

[27] Maher C., Feiner B., Baessler K., Christmann-Schmid C., Haya N., Brown J. (2016). Surgery for women with anterior compartment prolapse. Cochrane Database Syst. Rev..

[28] Holt E. (2019). US FDA rules manufacturers to stop selling mesh devices. Lancet.

[29] (2019). Department of Error. Lancet.

[30] Frick A.C., Barber M.D., Paraiso M.F.R., Ridgeway B., Jelovsek J.E., Walters M.D. (2013). Attitudes Toward Hysterectomy in Women Undergoing Evaluation for Uterovaginal Prolapse. Female Pelvic Med. Reconstr. Surg..

[31] Korbly N.B., Kassis N.C., Good M.M., Richardson M.L., Book N.M., Yip S., Saguan D., Gross C., Evans J., Lopes V.V. (2013). Patient preferences for uterine preservation and hysterectomy in women with pelvic organ prolapse. Am. J. Obstet. Gynecol..

[32] Lyatoshinsky P., Fünfgeld C., Popov A., Bezhenar V., Krutova V., Ulrich D., Umek W. (2019). Pelvic organ prolapse patients’ attitudes and preferences regarding their uterus: Comparing German- and Russian-speaking women. Int. Urogynecol. J..

[33] Van Ijsselmuiden M.N., Van Oudheusden A.M., Veen J., Van De Pol G., Vollebregt A., Radder C.M., Housmans S., Van Kuijk S.M., Deprest J., Bongers M.Y. (2020). Hysteropexy in the treatment of uterine prolapse stage 2 or higher: Laparoscopic sacrohysteropexy versus sacrospinous hysteropexy—A multicentre randomised controlled trial (LAVA trial). Int. J. Obstet. Gynaecol..

[34] Urdzík P., Kalis V., Blaganje M., Rusavy Z., Smazinka M., Havir M., Dudič R., Ismail K.M. (2020). Pelvic organ prolapse and uterine preservation: A survey of female gynecologists (POP-UP survey). BMC Women’s Health.

[35] Swift S.E. (2000). The distribution of pelvic organ support in a population of female subjects seen for routine gynecologic health care. Am. J. Obstet. Gynecol..

[36] DeLancey J.O. (2002). Fascial and muscular abnormalities in women with urethral hypermobility and anterior vaginal wall prolapse. Am. J. Obstet. Gynecol..

[37] Toozs-Hobson P., Boos K., Cardozo L. (1998). Management of vaginal vault prolapse. Int. J. Obstet. Gynaecol..

[38] (1995). Transvaginal sacrospinous colpopexy for vault and marked uterovaginal prolapse. Int. J. Gynecol. Obstet..

[39] DeLancey J.O. (1999). Structural anatomy of the posterior pelvic compartment as it relates to rectocele. Am. J. Obstet. Gynecol..

[40] Grimes C.L., Tan-Kim J., Nager C.W., Dyer K.Y., Menefee S.A., Diwadkar G.B., Overholser R.H., Xu R., Lukacz E.S. (2014). Outcome measures to assess anatomy and function of the posterior vaginal compartment. Int. Urogynecol. J..

[41] Grimes C.L., Lukacz E.S. (2012). Posterior vaginal compartment prolapse and defecatory dysfunction: Are they related?. Int. Urogynecol. J..

[42] Reena C., Kekre A., Kekre N. (2007). Occult stress incontinence in women with pelvic organ prolapse. Int. J. Gynecol. Obstet..

[43] Sinha D., Arunkalaivanan A.S. (2007). Prevalence of occult stress incontinence in continent women with severe genital prolapse. J. Obstet. Gynaecol..

[44] Haessler A.L., Lin L.L., Ho M.H., Betson L.H., Bhatia N.N. (2005). Reevaluating occult incontinence. Curr. Opin. Obstet. Gynecol..

[45] Engh A.M.E., Ekeryd A., Magnusson Å., Olsson I., Otterlind L., Tobiasson G. (2011). Can de novo stress incontinence after anterior wall repair be predicted?. Acta Obstet. Gynecol. Scand..

[46] Visco A.G., Brubaker L., Nygaard I., Richter H.E., Cundiff G., Fine P., Zyczynski H., Brown M.B., Weber A.M., Network P.F.D. (2008). The role of preoperative urodynamic testing in stress-continent women undergoing sacrocolpopexy: The Colpopexy and Urinary Reduction Efforts (CARE) randomized surgical trial. Int. Urogynecol. J..

[47] Chughtai B., Spettel S., Kurman J., De E. (2011). Ambulatory Pessary Trial Unmasks Occult Stress Urinary Incontinence. Obstet. Gynecol. Int..

[48] Pauls R.N., Segal J.L., Silva W.A., Kleeman S.D., Karram M.M. (2006). Sexual function in patients presenting to a urogynecology practice. Int. Urogynecol. J..

[49] Rogers R.G., Pauls R.N., Thakar R., Morin M., Kuhn A., Petri E., Fatton B., Whitmore K., Kingsberg S.A., Lee J. (2018). An international Urogynecological association (IUGA)/international continence society (ICS) joint report on the terminology for the assessment of sexual health of women with pelvic floor dysfunction. Int. Urogynecol. J..

[50] Maher C.F., Baessler K.K., Barber M.D., Cheong C., Consten E.C.J., Cooper K.G., Deffieux X., Dietz V., Gutman R.E., Van Iersel J.J. (2018). Surgical management of pelvic organ prolapse. Climacteric.

[51] Santis-Moya F., Pineda R., Miranda V. (2020). Preoperative ultrasound findings as risk factors of recurrence of pelvic organ prolapse after laparoscopic sacrocolpopexy. Int. Urogynecol. J..

[52] Diez-Itza I., Avila M., Uranga S., Belar M., Lekuona A., Martin A. (2020). Factors involved in prolapse recurrence one year after anterior vaginal repair. Int. Urogynecol. J..

[53] Medina C.A., Candiotti K., Takacs P. (2008). Wide genital hiatus is a risk factor for recurrence following anterior vaginal repair. Int. J. Gynecol. Obstet..

[54] Weemhoff M., Vergeldt T.F.M., Notten K., Serroyen J., Kampschoer P.H.N.M., Roumen F.J.M.E. (2011). Avulsion of puborectalis muscle and other risk factors for cystocele recurrence: A 2-year follow-up study. Int. Urogynecol. J..

[55] Oversand S.H., Staff A.C., Borstad E., Svenningsen R. (2018). The Manchester procedure: Anatomical, subjective and sexual outcomes. Int. Urogynecol. J..

[56] Wong V., Shek K., Rane A., Goh J., Krause H., Dietz H.P. (2013). Is levator avulsion a predictor of cystocele recurrence following anterior vaginal mesh placement?. Ultrasound Obstet. Gynecol..

